# How Accurate and Robust Are the Phylogenetic Estimates of Austronesian Language Relationships?

**DOI:** 10.1371/journal.pone.0009573

**Published:** 2010-03-10

**Authors:** Simon J. Greenhill, Alexei J. Drummond, Russell D. Gray

**Affiliations:** 1 Department of Psychology, University of Auckland, Auckland, New Zealand; 2 Computational Evolution Group, University of Auckland, Auckland, New Zealand; University of Miami, United States of America

## Abstract

We recently used computational phylogenetic methods on lexical data to test between two scenarios for the peopling of the Pacific. Our analyses of lexical data supported a pulse-pause scenario of Pacific settlement in which the Austronesian speakers originated in Taiwan around 5,200 years ago and rapidly spread through the Pacific in a series of expansion pulses and settlement pauses. We claimed that there was high congruence between traditional language subgroups and those observed in the language phylogenies, and that the estimated age of the Austronesian expansion at 5,200 years ago was consistent with the archaeological evidence. However, the congruence between the language phylogenies and the evidence from historical linguistics was not quantitatively assessed using tree comparison metrics. The robustness of the divergence time estimates to different calibration points was also not investigated exhaustively. Here we address these limitations by using a systematic tree comparison metric to calculate the similarity between the Bayesian phylogenetic trees and the subgroups proposed by historical linguistics, and by re-estimating the age of the Austronesian expansion using only the most robust calibrations. The results show that the Austronesian language phylogenies are highly congruent with the traditional subgroupings, and the date estimates are robust even when calculated using a restricted set of historical calibrations.

## Introduction

The past few years have seen a number of high-profile applications of Bayesian phylogenetic methods to lexical data [1], [2], [3] that have been very controversial [4]. The key topics of contention have been how accurate phylogenetic methods are at recovering linguistic history and how congruent the results are with the traditional linguistic comparative method. Recently we tested between scenarios of Pacific settlement by applying Bayesian phylogenetic methods to lexical data [3]. During the Holocene, a new culture–the Austronesians–arose in the Pacific and spread through the region from Taiwan, into Island South-East Asia and on to Oceania, eventually reaching as far afield as Madagascar, Hawaii, Rapanui, and New Zealand. There are two major competing hypotheses about the origins of the Austronesian peoples. The first hypothesis argues for an origin in Taiwan around 5,500 years Before Present (BP), followed by a “pulse and pause” style expansion through the Pacific into the Philippines and Island South-East Asia, along the coast of New Guinea and into Oceania [5], [6], [7], [8]. The second “slow boat” hypothesis argues for a much older origin in Island South-East Asia around 13,000–17,000 BP followed by a two-pronged expansion flowing north into Taiwan, and east into Oceania [9], [10], [11].

The genetic evidence for Pacific settlement is equivocal. Proponents of the “slow boat” hypothesis base their claims on mitochondrial studies that show high levels of genetic diversity in Island South-East Asia [10], [11] with estimated coalescence times ranging from 3,200 to 62,000 BP [10], [11], [12]. In contrast, evidence from Y chromosome and whole genome studies provide evidence for a pulse-pause type scenario of Taiwanese origins [13], [14], [15]. However, these inferences about Pacific prehistory drawn from genetic data have been hampered by problems separating ancient from recent admixture [16], and difficulties precisely dating the mitochondrial and Y chromosome haplogroups found in the Pacific due to systematic biases in rate variation over time [17], [18]. Moreover, the slow rate of molecular evolution in DNA makes it difficult to clearly resolve human prehistory during the Holocene, even in rapidly evolving molecules like mitochondrial DNA.

Languages are good markers of cultural groups [19]. As the Austronesian peoples spread throughout the Pacific the languages they spoke diversified into one of the largest language families in the world containing around 1,000 to 1,200 languages [20]. We recently applied computational phylogenetic methods to language data to test between the pulse-pause and slow boat scenarios of Pacific settlement [3]. The lexical data we used to test these hypotheses was drawn from the Austronesian Basic Vocabulary Database [21] which contains wordlists of 210 items of basic vocabulary that are thought to be stable over time and resistant to borrowing such as words for body parts, animals, kinship terms, simple verbs, colors, and numbers [21]. The homologous word forms in this database–cognates–were identified using the linguistic comparative method to identify systematic sound correspondences [4], [21]. In Gray et al [3] we encoded the cognate set information for 400 Austronesian languages into a binary form denoting cognate presence or absence in each language. We found the language phylogenies built from this data to be in striking accord with the pulse-pause scenario of Pacific settlement and incompatible with the slow-boat hypothesis.

The four central findings of our analysis were:

1. All the Formosan (aboriginal Taiwanese) languages were placed at the base of the language phylogenies.
2. The phylogenies had a “chained” topology consistent with a population expansion that started in Taiwan and then moved through to the Philippines, Borneo/Sulawesi, Central Malayo-Polynesia, South Halmahera/West New Guinea, and finally out into Oceania.
3. The age of the Austronesian language family was estimated to be approximately 5,200 years (95% highest posterior density interval, 4,750 to 5,800 years BP).
4. The branch lengths in the estimated phylogenies suggest a series of settlement pauses and expansion pulses.

However, there are two limitations in the analyses reported by Gray et al [3]. The first limitation was that we did not quantitatively assess the congruence between the subgroups identified in our language phylogenies and those identified by historical linguistics. We reported that our analyses supported 26 of the 34 main Austronesian subgroups proposed by historical linguists [20]. We argued that this showed striking congruence between our phylogenies derived from basic vocabulary, and the traditional subgroupings defined largely on the basis of phonological evidence such as the loss of the Proto-Oceanic uvular trill *R in the Central Pacific subgroup [22], or the lowering of high vowels in morphemes identifying Central-Eastern Malayo-Polynesian [23]. Despite this broad congruence, however, we have recently identified a relatively small number of languages where the traditional linguistic and Bayesian methods disagree. In total this affects 25 out of the 400 languages (See Materials and Methods). A tedious tactic adopted by some critics of language phylogenies [24], [25] is to point out a number of minor subgrouping issues and argue that this invalidates the entire tree topology. The misplacement of 25/400 languages might superficially suggest that the phylogenetic topologies and the subgroupings expected by historical linguistics are “wildly different”. However, with 400 languages there are 7.3×10^982^ possible rooted bifurcating trees [26]. This number of trees is vastly larger than the number of atoms in the universe. With finite amounts of data it is simply not realistic to expect to accurately estimate every single branching point in a tree of 400 languages. Some lack of resolution and minor misplacement of languages is to be expected even with very large datasets and very good models. Rather than focusing on individual languages, a quantitative analysis of the overall degree of congruence gives a more accurate assessment of the similarity between different phylogenies. For this reason, phylogeneticists have developed a suite of tree comparison metrics to systematically compare trees and quantify their differences [27], [28].

The second limitation of Gray et al [3] concerns the robustness of the date estimates for proto-Austronesian. To estimate the age of Proto-Austronesian we used a method known as penalized likelihood rate-smoothing [29]. This method smoothes the observed rates of lexical change over the branches of the language phylogeny, whilst incorporating date information from calibration points. In Gray et al [3] we used a combination of 14 archaeological and historical dates to calibrate our trees. Our results placed the age of the Austronesian expansion at around 5200 B.P.–strikingly congruent with the time-depth expected by the pulse-pause scenario. These calibrations included archaeological information about when parts of the Pacific were settled by Austronesian-speaking peoples (e.g. Oceania, Madagascar, Eastern Polynesia), historical information about when the original linguistic data was collected (e.g. around 350 years ago for the Favorlang and Siraya languages), or attestation in other historical records (e.g. Chinese records mentioning the Chamic language subgroup around 1,800–2,500 BP [30]). However, we did not emphasise how robust these date estimates were to calibration error.

In this paper we address these two limitations. First, we use quantitative tree comparison metrics to evaluate the congruence between the Austronesian language phylogenies and the evidence from historical linguistics. Second, we re-date the Austronesian language phylogenies using only the most robustly attested historical information.

## Results

**Figure 1:**
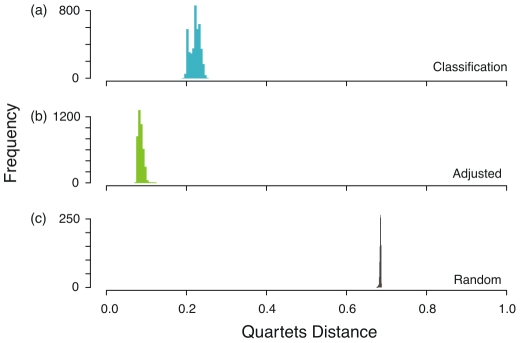
Histogram of quartet distances for the classification tree (1a), the adjusted maximum clade credibility tree modified to the expected linguistic topology (1b), and a randomized tree distribution (1c).

To quantify the differences between the language phylogenies and the linguistic classification, we calculated the quartets distance between the 4,200 trees in the posterior probability distribution presented in Gray et al [3] and the tree derived from the classification information in the Ethnologue [31]. The median normalized quartets distance between the Ethnologue classification and the posterior tree distribution was 0.223 (s.d. = 0.012). As an alternative comparison, we also quantified the distance between the Gray et al trees and the tree adjusted to address the language subgrouping issues we identified. The median quartets distance here was 0.085 (s.d. = 0.006). In contrast the distance between the posterior tree distribution and the randomized distribution of tree topologies is much larger with a median of 0.685 (s.d. = 0.002). Figure 1 shows histograms of these three distributions.

**Figure 2:**
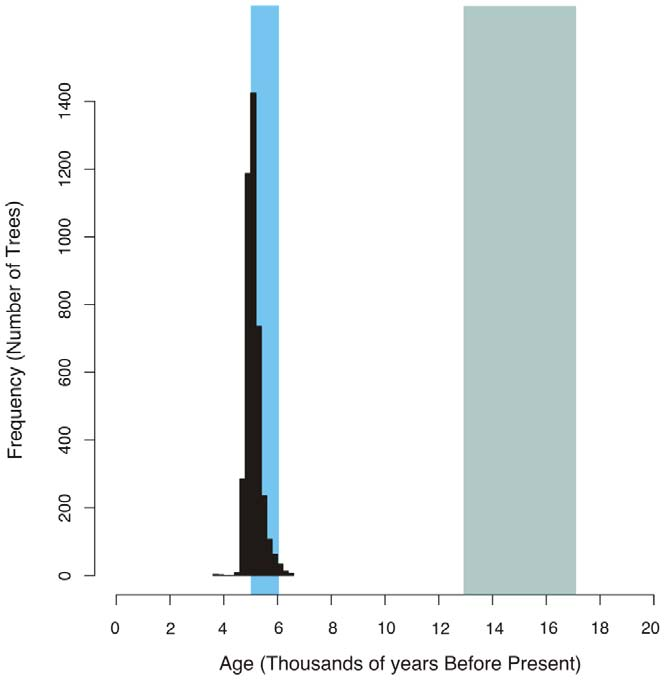
Histogram of the estimated age of the Austronesian language family calculated using a conservative set of calibration points. The light blue bar shows the age range predicted by the pulse-pause scenario (5,000 to 6,000 years BP), and the gray bar shows that predicted by the slow-boat scenario (13,000 to 17,000 years BP). The mean age estimate for Proto-Austronesian is 5,117 BP.

We recalculated the age of the Austronesian language family using only the most robust calibration information. The estimated age of the trees had a mean of 5,117 years, and a 95% highest posterior density interval of 4,660 to 5,680 years BP (Figure 2). Despite the much fewer calibrations used in this re-analysis, we find date estimates consistent with those presented in Gray et al [3], and consistent with a recent Taiwanese origin of the Austronesian peoples as predicted by the pulse-pause scenario.

## Discussion

### Congruence of Topology

First, we compared the 4,200 trees from the posterior probability distribution presented in Gray et al to the language classification in the Ethnologue [31]. If the language phylogenies were close to the Ethnologue classification then the quartets distance will be small and close to 0.0. However, if the language phylogenies and the expected classification tree are very different, then the quartets distance will be closer to 1.0. The mean quartets distance between the Ethnologue classification tree and the phylogenies was 0.223 (s.d. = 0.012, Figure 1a). This value of 0.223 is low, showing that the trees from Gray et al are indeed close to the expected classification tree.

It could be argued that the Ethnologue classification may not be the best comparison because the classification tree is highly unresolved, contains over-estimates of language diversity, and the classification often understandably lags behind linguistic research [32], [33]. Therefore, we made a second comparison by inspecting the Gray et al trees to identify languages that were incorrectly placed. We identified 25 languages in the phylogenies that were not subgrouped in accordance with the linguistic evidence (See Materials and Methods). We then adjusted the maximum clade credibility tree to match these expected subgroupings. The quartets distance between this tree and the posterior tree distribution was again very low with a median of 0.085 (s.d. = 0.006). To characterize the obtained quartets score more fully, we calculated the quartets distance between 1000 random trees and the maximum clade credibility tree from the posterior tree distribution (Figure 1c). The quartets distance to the randomized trees had a median of 0.685 (s.d. = 0.002)–much larger than both the quartets scores for the classification and adjusted trees.

**Figure 3:**
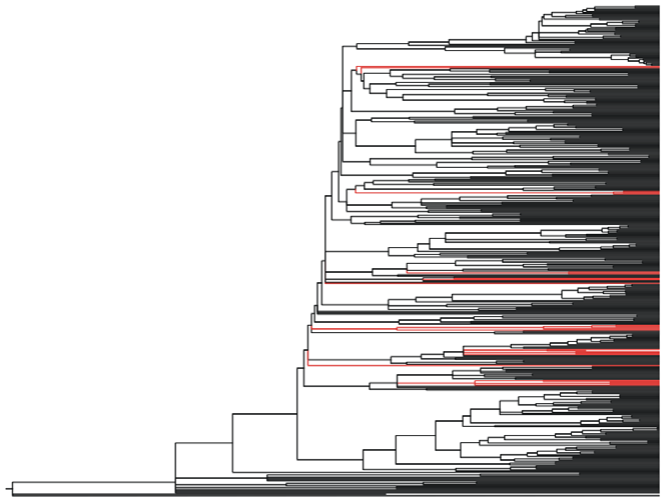
Maximum clade credibility tree showing the branches belonging to the 25 languages that differ between the language phylogenies and the expected classification according to historical linguistics. The queries concern only a small number of languages and do not affect the rooting and chain-like expansion sequence.

If the Gray et al [3] trees were not showing strong congruence with the tree topologies predicted by linguistic evidence, then the quartets distance between them and the classification or adjusted topologies would be large–and approaching that of the random distribution of tree topologies. Instead, the quartets results show that the differences between the Gray et al [3] tree topologies, and the subgroupings proposed by linguistics are relatively small. How might these small differences have arisen? There at least three possible causes for these differences. First, any analysis contains statistical error due to the data chosen and the sampling method used. Some of the 25 misplacements we identified might be due to lack of data–our analyses used basic vocabulary, but many language subgroups are defined not by lexicon but by shared innovations in phonology or morphology. Second, there will be error due to model misspecification. In Gray et al [3] we compared a number of different models and used the best performing one. However, the fundamental nature of a model is to simplify reality, and there will always be some degree of misspecification [34]. In our data, the major culprit of model misspecification is likely to be linguistic borrowing–this is probably the cause of at least 21/25 misplacements (See Materials and Methods). Language borrowing is often cited as a major problem for phylogenies of languages [35], [36], but in a recent paper we show that inferences made with phylogenetic methods (such as the estimated ages of the common ancestors) are robust to realistic levels of language borrowing and diffusion [37]. Whilst borrowing may cause slight disruptions of the lower-level topology, the misplacements do not affect our central findings about the Taiwanese rooting and chain-like expansion sequence revealed in our trees (Figure 3).

The third possible reason for the differences between our Austronesian language phylogenies and the traditional subgroupings is that our phylogenetic estimates may be recovering relationships where the traditional subgroupings are over-confident. For example, one of the 25 misplaced languages, Irarutu, has been problematic for some time (e.g. [23], [38], [39]. Blust states that “Irarutu apparently is not a (Central Malayo-Polynesian) language, and shows no known positive evidence of belonging to the (South Halmahera/West New Guinea) group. Its position for the present remains indeterminate” ([40], p.272). Current opinion weakly subgroups Irarutu with South Halmahera/West New Guinea, possibly as a first-order subgroup [39]. Our analyses are reflecting this classificatory difficulty by placing Irarutu between the Central Malayo-Polynesian and South Halmahera/West New Guinea languages. The language phylogenies appropriately represent classification difficulties with larger groups too. For example the phylogenies do not recover two previously proposed subgroups: Western and Central Malayo-Polynesian. Western-Malayo-Polynesian used to be considered one of the major subsets of Austronesian, however recent opinion holds that these languages actually form a number of primary branches within Malayo-Polynesian [20]. Central Malayo-Polynesian is a dialect linkage with low internal cohesion and is identified only by innovation-linked overlapping isoglosses [23]. Therefore, rather than being incorrect, the Bayesian language phylogenies are reflecting this uncertainty in the language subgrouping.

### Robustness of Dating

The second core claim of Gray et al [3] was that the age of the Austronesian population expansion dated to around 5,200 years B.P. This was consistent with the “pulse-pause” scenario of Pacific origins, and contradicted the alternative “slow-boat” scenario that proposed a much older origin around 13,000–17,000 BP. However, the dating method we used relied on a number of archaeological calibration points that could be contested. For example, one calibration we used linked the appearance of red-slipped pottery in the Philippines to the arrival of the Austronesians in that region [5], [41], [42].

To evaluate the robustness of the dating in Gray et al[3], we re-estimated the age of the Austronesian expansion under a heavily reduced set of calibrations. The first four calibrations were the historical ages when source data was collected for Old Javanese (700–1,200 BP), Old Chinese (2,300–2,9001 BP), Favorlang (346–384 BP) and Siraya (346–384 BP). The fifth calibration Proto-Oceanic (3,200–3,6001 BP), is based on a wealth of evidence linking the entry of the Proto-Oceanic society to the appearance of the Lapita cultural complex in Near Oceania. The re-dated Austronesian language phylogenies again clearly support the pulse-pause scenario of Pacific settlement over the slow boat scenario with an estimated origin around 5,100 years BP (Figure 2).

One could claim that attempting to link languages to cultures to archaeology is fraught with problems [43]. Just as a person who drives a Volvo is not necessarily Swedish, pottery can be traded independently of language and therefore might not be a reliable calibration. However, the evidence linking Lapita to Proto-Oceanic is much stronger than a single cultural artifact. The intrusion of the Lapita cultural complex into Near Oceania brought a marked shift in cultures from the non-Austronesian societies to the Austronesian-style agricultural society. Lapita society was not only agricultural, but many of the common food plants and domesticated animals can be traced back to Southeast Asia origin [44], [45], [46]. The social organisation of Lapita was distinctively Austronesian [47], [48]. Many Lapita characteristics can be reconstructed in the Proto-Oceanic (POc) lexicon [44], [49]. This includes, for example, linking the Lapita adze/axe kits to Proto-Oceanic *kiRam or *matau [50], the linking of housing types to e.g. POc *Rumaq [46], reconstructions of fishing equipment like the one-piece rotating fishhooks, and one-piece trolling lure (POc *kawil and *bayan respectively, [51]), and terminology for the Malayo-Polynesian outrigger canoe sailing complex [52]. Driving a Volvo does not make one a Swede; however, if you also eat distinctively Swedish cuisine, live in a distinctively Swedish-type society, and have a wide collection of Swedish cultural artifacts, then there is a very high probability that you are indeed Swedish.

### Conclusion

In this paper we have addressed two potential limitations with Gray et al [3]. First, we have reassessed the congruence between the language phylogenies and the subgroups expected by the linguistic comparative method. Our results show that the Austronesian basic vocabulary trees are strikingly congruent with traditional language subgroups proposed mainly on the basis of phonological innovations by historical linguists. Second, we have re-estimated the age of Proto-Austronesian using a more restricted set of calibrations. The new age estimates are consistent with those presented in Gray et al [3] in supporting a pulse-pause population expansion around 5,200 years ago from Taiwan.

## Materials and Methods

### Topological Differences

The language trees presented in Gray et al [3] show a broad consensus with the overall language subgroupings proposed by historical linguistics [20]. However, we identified 25 instances of language placements in the Gray et al [3] results that were not in accordance with linguistic evidence. In the Oceanic subfamily, the Willaumez languages (Nakanai, Maututu, Lakalai) are placed with the North New Guinea languages but instead belong to the large Meso-Melanesian subgroup. This placement is possibly due to unidentified lexical borrowings between these Willaumez languages and the neighboring languages of West New Britain belonging to the Meso-Melanesian subgroup. Second, the language Mussau is linked to the base of the Meso-Melanesian subgroup, followed by the language Vitu. The placement of Vitu at the base of this subgroup is not particularly surprising given that the Bali-Vitu lineage is thought to be a primary branch of Meso-Melanesian [22]. However, Mussau is the only extant member of the Saint Matthias subgroup [53] and therefore should be placed as a higher order subgroup inside Oceanic, and not inside Meso-Melanesian. Deeper in the tree, the language Irarutu (aka Kasira) belongs to the South Halmahera/West New Guinea subgroup [38], but in our results, this language falls to the base of the parent clade (Eastern Malayo-Polynesian, [38]).

The Gray et al [3] results also show some incongruencies with the Central Malayo-Polynesian linkage. First, two languages of Aru (Ujir and Ngaibor) are placed as a sister group to the Central Maluku languages. Current linguistic opinion places both the Aru and Central Maluku subgroups as subgroups of the Central Malayo-Polynesian subgroup with no known links between them. Second, the Gray et al [3] trees weakly place Koiwai and Kei inside the Yamdena-North Bomberai group. Kei is a member of the Southeast Maluku subgroup, whilst Koiwai is a member of the closely neighboring subgroup of South Bomberai [23]. These results could suggest a greater subgroup including the Yamdena-North Bomberai with the South Bomberai languages. Alternatively, the placement of Koiwai here may reflect the widespread diffusion of features such as glide truncation across the Bomberai region [23]. On first glance, the placement of Kei with these languages is unusual. However, Blust (personal communication, 17/3/2009) has unpublished data suggesting that Kei probably belongs to a slightly larger group that includes Yamdena-North Bomberai as indicated by the Gray et al results.

The Western Malayo-Polynesian linkage also shows a number of misplaced languages in the Gray et al [3] results. First, the language Maloh is not included in the Greater South Sulawesi group, but instead falls to the base of the parent clade. Second, our trees grouped the Barito languages with the North Borneo subgroup. However, the most likely sister-clade for the Barito languages is the Sama-Bajaw languages (R. Blust, personal communication, 17/3/2009). We have been reassessing the cognate coding in that area, and have uncovered 17 previously unrecognised loan words in the Sama-Bajaw language Inabaknon. These borrowings are the likely explanation for the minor mismatch between our results and the traditional linguistic subgroupings in this region. Third, the Sangiric language subgroup is placed as a higher-order grouping within the Western Malayo-Polynesian languages. The Sangiric languages are located in the Sulawesi region but should be a primary branch of the Philippines family [54], [55]. Our placement of Sangiric as a deeper group within the Western Malayo-Polynesian linkage may either reflect contact-induced change with neighboring Sulawesi languages, or it may reflect the repeated parallel drift that has occurred in Sangiric and other Sulawesi-area languages [55].

Finally, the Malayo-Sumbawan subgroup inferred by Gray et al [3] differs to that proposed by Adelaar [56], [57] by including Javanese, and the Sumatran languages (e.g. Lampung, Gayo or Batak). In our original paper we suggested that these differences might be explained by unidentified borrowings between languages within these subgroups. For example, Balinese has a number of vocabulary registers and the higher status register is heavily Javanised [56]. It is possible that the Balinese word list reflects this Javanised register that may have caused the Javanese language to be placed inside this subgroup (M. Ross, personal communication, 22/12/2008).

### Quantifying Topological Similarity

We used a standard tree-comparison metric, the quartets distance [27], [28], [58], to quantify how congruent the Gray et al [3] tree was with the traditional linguistic subgroupings. The quartets distance measures the number of different combinations of four language subsets in both trees. The normalized quartets score is obtained by dividing by the total number of quartets for the tree. The normalized score will range from 0.0 for identical trees to 1.0 for maximally different tree topologies.

First, we modified the Gray et al [3] maximum clade credibility tree (which is a single tree summary of the posterior tree distribution) to match the above subgrouping issues. This provided us with a tree topology “adjusted” to match the expected linguistic evidence. Second, to act as an alternative classification tree, we constructed a “classification” tree from the language subgrouping information in the Ethnologue online [31]. We then systematically calculated the normalized quartets distance between the “classification” and “adjusted” trees to each of the 4,200 trees in the Gray et al [3] posterior probability distribution. To provide a comparison of the obtained quartets scores for the adjusted and classification trees we randomly generated 1,000 trees using PAUP* v4.b10 [59]. We then calculated the quartets distance from the maximum clade credibility tree to each of these random trees.

### Phylogenetic Dating

To assess the robustness of the timing inferences we reanalyzed the age of the Austronesian expansion on the language phylogenies presented in Gray et al [3]. In this paper, we presented posterior probability distributions of language trees calculated under three different models of language evolution. The single-rate model with covarion fit the data better (Bayes Factor = 1034) than a two-rate model with gamma-distributed rate heterogeneity, therefore we follow Gray et al [3] in selecting this as the primary analysis. We selected the 4,200 trees from the posterior probability distribution of the single-rate model of cognate gain and loss.

To assess the robustness of the date estimates on the Austronesian language phylogenies we used the 5 least controversial calibrations.

1. Proto-Oceanic (3,200–3,600 BP).
2. Old Javanese (700–1,200 BP).
3. Old Chinese (2,300–2,900 BP).
4. Favorlang (346–384 BP).
5. Siraya (346–384 BP).

The first calibration, Proto-Oceanic, is linked by many threads of evidence to the Austronesian entry into Near Oceania (see main text). The other four calibrations are the historical dates at which those specific languages were collected.

The age of the Austronesian expansion was then estimated using these calibrations on all 4,200 trees in the posterior using a penalized likelihood rate-smoothing approach implemented in the program *r8s* v1.71 [29], [60]. This method converts the obtained branch-lengths into time estimates by smoothing the rates of change over the tree according to the calibration information.
